# HilA-like regulators in *Escherichia coli* pathotypes: the YgeH protein from the enteroaggregative strain 042

**DOI:** 10.1186/s12866-014-0268-5

**Published:** 2014-10-25

**Authors:** Mário Hüttener, Manuela Dietrich, Sònia Paytubi, Antonio Juárez

**Affiliations:** Institute for Bioengineering of Catalonia (IBEC), Barcelona, Spain; Department of Microbiology, Faculty of Biology, University of Barcelona, Barcelona, Spain

**Keywords:** HilA, YgeH, *E. coli* 042, H-NS

## Abstract

**Background:**

The HilA protein is the master regulator of the *Salmonella* pathogenicity island 1 (SPI1). EilA and YgeH proteins show a moderate similarity to HilA and are encoded in pathogenicity islands from several *E. coli* strains, both pathogenic and non-pathogenic. In the present work we characterize the YgeH protein from the enteroaggregative *E. coli* strain 042 (locus tag EC042_3050).

**Results:**

We show that both *E. coli* 042 YgeH and EilA proteins are able to functionally replace HilA in *Salmonella*. Interestingly, this is not the rule for all YgeH proteins: the YgeH protein from the enterohaemorragic *E. coli* strain O157 appears to be non-functional. *ygeH* expression is not influenced by growth osmolarity or temperature, and moderately increases in cells entering the stationary phase. H-NS represses *ygeH* expression under all growth conditions tested, and binds with specificity to the *ygeH* promoter region. As expected, expression of ETT2 (*Escherichia coli* type 3 secretion system 2) genes requires YgeH: ETT2 operons are downregulated in a *ygeH* mutant. Accordingly, since H-NS represses *ygeH* expression, ETT2 expression is significantly increased in an *hns* mutant.

**Conclusion:**

*E. coli* 042 YgeH protein is functional and able to replace HilA in *Salmonella*. ETT2 gene expression requires YgeH activity which, in turn, is subjected to H-NS silencing.

**Electronic supplementary material:**

The online version of this article (doi:10.1186/s12866-014-0268-5) contains supplementary material, which is available to authorized users.

## Background

Diarrhoeal disease is the second leading cause of infant mortality under the age of 5 worldwide [1]. Enteropathogenic *Escherichia coli* (EPEC) is a human pathogen of the small intestine and is a significant cause of infantile diarrhea [2]. EPEC were recognized as pathogens several decades ago [3]. Later on, EPEC and enteroaggregative *E. coli* (EAEC) were distinguished from each other on the basis of their patterns of adherence to HEp-2 cells. Unlike the localised “microcolonyforming” pattern of adherence associated with EPEC, EAEC display a characteristic aggregative or “stacked-brick” pattern of adherence [4]. Nataro *et al.* demonstrated a significant association of EAEC with diarrhea in a case control study of children in Chile [4]. Immediately following the discovery of EAEC as a category of pathogenic *E. coli*, several epidemiological reports casted doubt on the pathogenic nature of EAEC [5]. Nevertheless, a volunteer study provided evidence for EAEC strain 042 eliciting diarrhea in the majority of volunteers [6]. Since then many studies have supported the association of EAEC and diarrhea in both developing countries and industrialized nations. Thus, EAEC have been significantly associated with (i) endemic diarrhea in infants in developing and industrialised nations, (ii) persistent diarrhea in HIV-positive patients, (iii) traveller’s diarrhea, (iv) food/ water-borne outbreaks, and (v) sporadic cases of diarrhea [6]. Furthermore, the increasing number of reports in which EAEC is implicated as the agent mediating diarrhea suggests that *E. coli* strains belonging to this pathotype are important emerging pathogens [7]. A large number of virulence factors has been associated with clinical illness in epidemiologic studies [8]. It is also remarkable that EAEC strains are heterogeneous [9]. As not all strains of EAEC tested elicited diarrhea, the EAEC strain 042 which caused diarrhea in the volunteer study became the prototypical EAEC strain for the study of virulence factors and EAEC pathogenicity [6]. Its genome has been sequenced [10] and the virulence factors characterized.

Success for a bacterial pathogen to cause disease requires not only the display of several virulence determinants, but also a precise control of their expression, such that each is expressed at the appropriate time and place in the host. A good example for that is expression of the *Salmonella* pathogenicity island 1 (SPI1). Several environmental factors and regulators have been identified as affecting SPI1 [11]. It has been shown that SPI1 environmental regulation converges in the modulation of the *hilA* gene [12]. *hilA* is located on SPI1 and encodes the HilA protein, a member of the OmpR/ToxR family of regulators [13,14]. HilA regulation itself is very complex and responds to several stimuli [15–17]. Both positive and negative regulators dictate appropriate HilA levels, which, in turn, result in activation or repression of the SPI-encoded effectors.

A genomic analysis of the type 3 secretion system (TTSS) from *E. coli* showed that two different genomic islands encoded *hilA* homologues [18]. The *eip* island, identified in the *E. coli* 042 genome encodes, in addition to different effectors, the *eilA* gene, a HilA-like regulator [19]. In addition, the ETT2 pathogenicity island encodes the *ygeH* gene, which shows significant similarity to HilA. This latter pathogenicity island also encodes a TTSS and different effectors. Remarkably, whereas ETT2 appears to be functional in *E. coli* 042, it has been subjected in the rest of the strains analysed to varying degrees of mutational attrition that results in a non-functional TTSS.

The role of EilA, the HilA-homologue encoded in the genome of the enteroaggregative strain 042 was characterized a few years ago [19]. EilA expression was reported to modulate expression of different genes of the *eip* island (i.e., *eipB, eipC, eipD, eicA* and *eaeX*), as well as of two genes of a pathogenicity island not linked to *eip* (ETT2). EilA defect was associated to alterations in adherence to epithelial cells and to biofilm formation. A role for EilA modulating expression of the TTSS and effectors of different chromosomal islands in EAEC was suggested. In contrast to EilA, the regulatory role of YgeH has not been hitherto characterized. We study in this report YgeH. We show that it is able to compensate for HilA depletion in *Salmonella*. We also study its regulation, and describe some of its target genes in the ETT2 pathogenicity island from strain 042.

## Methods

### Bacterial strains, plasmids and culture conditions

All bacterial strains and plasmids used in this study are listed in Table 1. Cultures were normally grown in Luria-Bertani (LB) medium (10 g NaCl, 10 g tryptone and 5 g yeast extract per litre) at the indicated temperature with vigorous shaking at 200 r.p.m. (Innova 3100, New Brunswick Scientific). Where indicated, LB medium without NaCl (0 g per liter), or D-MEM supplemented 1:1 with LB together with added 0.2% glucose (*eilA* induction conditions described by [19]) was used. Antibiotics were used at the following concentrations: kanamycin (50 μg ml^−1^), carbenicillin (100 μg ml^−1^) and chloramphenicol (25 μg ml^−1^).

**Table 1:** **Bacterial strains and plasmids used in this study**

**Strain or plasmid**	**Description**	**Source or reference(s)**
***S. enterica*** **serovar Typhimurium**		
SV5015	SL1344 *his*+	J. Casadesús
MH-C1	SV5015 *hilA*::Cm	[20]
MHS-1	SV5015 ∆hilA	[20]
SV5258	SV5015 Φ*invF-lacZY*	J. Casadesús
SV5258HilA	SV5258 ∆*hilA*::Cm	This work
MHS-2	SV5015 *sipA*::3XFLAG	This work
MHS-3	MHS-1 *sipA*::3XFLAG	This work
LB5000	rLT^−^ rSA^−^ rSB^−^	[21]
***Escherichia coli***		
*E. coli* O157:H7	does not produce either Shiga-like toxin I or II (ATCC N° 43888)	ATCC
AAG1	MG1655 ∆*LacZYA*	[22]
AAG1H	AAG1 ∆*hns*	This work
AAG1IHFA	AAG1 ∆*ihfA*::Cm	This work
AAG1IHFB	AAG1 ∆*ihfB*::Cm^−^	This work
AAG1HA	AAG1 ∆*hns* ∆*ihfA*::Cm	This work
AAG1HB	AAG1 ∆*hns* ∆*ihfB*::Cm	This work
AAG1λRS*ygeH*	AAG1 λRS*ygeH* integrated *attB* Km^r^	This work
AAG1HλRS*ygeH*	AAG1H + λRS*ygeH*	This work
AAG1HAλRS*ygeH*	AAG1HA + λRS*ygeH*	This work
AAG1HBλRS*ygeH*	AAG1HB + λRS*ygeH*	This work
EAEC 042	Cm^r^ Sm^r^ Tc^r^	I. Henderson
042HNS	EAEC 042 Δ*hns*	This work
042YgeH	EAEC 042 Δ*ygeH*	This work
042YgeH3X	EAEC 042 *ygeH*::3xFLAG	This work
042YgeH3XHNS	042YgeH3X Δ*hns*	This work
**Plasmids**		
pKD4	*bla* FRT *ahp* FRT PS1 PS2 oriR6K Km^r^ Cb^r^	[23]
pKD3	*bla* FRT *cat* FRT PS1 PS2 oriR6K Cm^r^ Cb^r^	[23]
pKD46	*bla* P_BAD_ *gam bet exo* pSC101 oriTS Cb^r^	[23]
pCP20	*bla cat cI*857 *l*P_R_ *flp* pSC101 oriTS Cb^r^	[24]
pSUB11	3xFLAG- and Km^r^-coding template vector	[25]
pBAD18	rep_pMB1_ p_*araBAD*_ Cb^r^	[26]
pBADHilA	pBAD18 + *hilA*_SV5015_	This work
pBADEilA	pBAD18 + *eilA*_EAEC042_	This work
pBADYgeH042	pBAD18 + *ygeH*_EAEC042_	This work
pBADYgeHO157	pBAD18 + *ygeH*_O157:H7_	This work
pRS551	Promoterless vector *lacZ* Km^r^ Cb^r^	[27]
pYgeH042	*ygeH*_042_ promoter cloned into pRS551	This work

To construct plasmids pBADEilA, pBADYgeH_042_, pBADHilA and pBADYgeH_O157_, *eilA* and *ygeH* genes of strain *E. coli* 042, *hilA* gene of *S.* Typhimurium SL1344 strain and *ygeH* gene from *E. coli* O157H7 were amplified using oligonucleotides EILAKPNI5-EILAHINDIII3, YGEHKPNI5-YGEHHINDIII3, HILABADFW-HILABADRV and YGEHO157XbaFW-YGEHO157HindRV, respectively (see Additional file 1). The oligonucleotides add *Kpn*I and *Hind*III sites, to the *eilA and ygeH* genes from *E. coli* 042, *Eco*RI and *Xba*I sites to the *hilA* gene and *Xba*I – *Hind*III sites to *ygeH* gene from *E. coli* O157:H7. The corresponding *Kpn*I-*Hind*III, *Eco*RI-*Xba*I and *Xba*I-*Hind*III PCR fragments were cloned into pBAD18 digested with the same enzymes, resulting in plasmids pBADEilA, pBADYgeH_042_, pBADHilA and pBADYgeH_O157_, respectively.

### Genetic manipulations

Standard molecular and genetic procedures were performed as described by [28]. Enzymes were used according to the manufacturer’s recommendations. Introduction of plasmids into *Escherichia coli* and *S.* Typhimurium strains was performed by electroporation of 10% glycerol-washed cells using an Eppendorf gene pulser (Electroporator 2510). Plasmids isolated from *E. coli* were first passaged through the restriction-deficient *S.* Typhimurium strain LB5000 [21] before transformation of *S.* Typhimurium SL1344 competent cells.

Chromosomal deletions of *hilA* from *S.* Typhimurium, *hns*, *ihfA* and *ihfB* from *E. coli* AAG1 and *ygeH* and *hns* from *E. coli* 042 were obtained by the λ Red recombinant method as previously described [23]. The antibiotic-resistance determinant of plasmid pKD4 was amplified using oligonucleotides HNSP1/HNSP2, YGEHP1/YGEHP2 for *hns* and *ygeH* genes, and, in the case of *hilA*, *ihfA* and *ihfB* deletions, the chloramphenicol resistance was amplified using as template the pKD3 plasmid and the oligonucleotides HIlAP1/HILAP2 and IHFAP1/IHFAP2, IHFBP1/IHFBP2 (see Additional file 1). Mutants were selected on LB plates containing the appropriate selection marker, and the successful deletion of the gene was confirmed by PCR using the primers CAT-C1 and CAT-C2 (chloramphenicol resistance; Cm^r^) or KT and K2 (kanamycin resistance; Km^r^) in combination with specific primers located in the remaining gene sequence nearby (Additional file 1, P1UP/P2DOWN series oligonucleotides). When necessary, the antibiotic resistance was then eliminated by transforming the mutant strain with plasmid pCP20 and subsequent incubation at 43°C for two passages according to [23]. The double deletions were obtained by combining one previously deletion with other deletion associated with an antibiotic resistance.

Chromosomal insertions of 3XFLAG sequences to the *sipA* and *ygeH* genes were obtained by a modification of the λ Red recombinant method, as described by [25]. The antibiotic-resistance determinant of plasmid pSUB11 was amplified using oligonucleotides SIPA3XP1/SIPA3XP2 and YGEH3XP1/YGEH3XP2 for *sipA* and *ygeH*, respectively (Additional file 1). Mutants were selected on LB plates containing kanamycin, and successful 3XFLAG insertion was confirmed by PCR using the oligonucleotides KT and K2 (kanamycin resistance; Km^r^) in combination with specific oligonucleotides located in the remaining gene sequence nearby (Additional file 1, 3XP1UP/3XP2DOWN series oligonucleotides). The SV5015SipA strain was used as a donor strain to transfer the SipA::3xFLAG fusion to strain SV5015HilA using phage P22 HT [29], generating strain SV5015SH. The chromosomal fusion YgeH::3xFLAG was constructed in the parental strain *E. coli* 042 and the isogenic mutant Δ*hns*, generating 042YgeH3X and 042YgeH3XHNS strains, respectively.

### Construction of an *ygeH::lacZ* transcriptional fusion

A transcriptional *ygeH::lacZ* fusion was constructed by cloning the promoter region of the *ygeH* gene from EAEC 042 into the pRS551 plasmid. A single copy of the fusion was then inserted into the chromosome of the AAG1 strain by using the bacteriophage λRS45 as described by [27]. First of all, the promoter region of the *ygeH* gene was amplified using the oligonucleotides YGEHBAMHI5/YGEHBAMHI3 (Additional file 1) that add a *Bam*HI site, and the resulting PCR product was purified using the kit High Pure PCR Product Purification Kit (Roche). Subsequently, the purified PCR product was digested with *Bam*HI enzyme (Fermentas) and ligated into the *Bam*HI-digested pRS551, generating the plasmid pYgeH. The transcriptional fusion *ygeH::lacZ* cloned into the plasmid pYgeH was then transferred to the λRS45 bacteriophage according to [27] and integrated as a single copy into the *attB locus* of the AAG1 chromosome. To confirm the correct insertion from the selected resulting colonies, a PCR screening was performed according to [30].

### Beta-galactosidase assay

β-galactosidase activity measurements were performed as described by [31].

### Cell-free supernatant preparation

The supernatants from cultures were obtained utilizing trichloroacetic acid (TCA) as agent for protein precipitation and acetone for removal traces from TCA treatment. The cultures were grown in LB medium until O.D._600nm_ at 2.0, and then 10 ml were centrifuged at 6.000 rpm for 10 minutes at room temperature. The supernatant was filtered with a 0.22 μm pore size filter (Millex GP, Millipore) and proteins were precipitated by adding TCA at a final concentration of 10% and maintaining samples on ice for 1 hour. The samples were then centrifuged for 30 minutes at 13.400 rpm at 4°C, the supernatants were discarded and 0.5 ml of acetone was added. The samples were centrifuged once more as described above. After removal of acetone the pellet was air-dried for 10 minutes and the samples were reconstituted in sample buffer (Laemmli sample buffer, Bio-Rad).

### SDS-PAGE and western blotting

Protein samples were analysed by SDS-PAGE at 10% and 12.5%. Proteins were transferred from the gels to PVDF membranes. Western blot analysis was performed with monoclonal antibodies raised against FLAG-epitope (1:10.000, Sigma) or against GAPDH (1:2000, Thermo Scientific) and horseradish peroxidase-conjugated goat anti-mouse IgG (1:2500, Promega). Detection was performed by enhanced chemiluminescence using Quantity One software (Bio-Rad).

### RNA isolation

Total RNA was extracted from bacteria using the RNeasy Mini kit (Qiagen) according to the manufacturer’s instruction. Potential traces of DNA were removed by digestion with DNase I (Turbo DNA-free, Ambion), according to the manufacturer’s protocol. RNA concentration and RNA quality were measured using a Nano- Drop 1000 (Thermo Fisher Scientific).

### Real-time qRT-PCR

Real-time quantitative reverse transcription-PCR (qRT-PCR) was performed as previously described [32]. Briefly, 1 μg of total RNA was reverse transcribed to generate cDNA using the High-capacity cDNA Reverse Transcription kit (Applied Biosystems) as recommended by the manufacturer. As a control, parallel samples were run in which reverse transcriptase was omitted from the reaction mixture. Oligonucleotides complementary to the genes of interest were designed using primer3 software. Real-time PCR using SYBR green PCR master mix (Applied Biosystems) was carried out on the ABI Prism 7700 sequence detection system (Applied Biosystems). After analysis of amplification plots with the ABI Prism sds software package, relative quantification of gene transcription was performed using the comparative threshold cycle (CT) method. The relative amount of target cDNA was normalized using the *gapA* gene as an internal reference standard.

### Electrophoretic mobility shift assay (EMSA)

For EMSA, a 228 bp fragment corresponding to the promoter region of the *ygeH* operon (nucleotides −207 to +21) was amplified using oligonucleotides YGEHBAMHI5/YGEHBAMHI3 (Additional file 1). For each reaction, 50 ng DNA were mixed with increasing concentrations of the purified H-NS-His protein in binding buffer (250 mM HEPES, pH 7.4, 350 mM KCl, 5 mM EDTA, 5 mM DTT, 500 μg BSA ml^−1^, 25% glycerol). After incubation for 30 min at room temperature, 20 μl of samples were separated on 5% polyacrylamide/0.5× TBE gel. The bands were stained using ethidium bromide and visualized using Quantity One software (Bio-Rad). For competitive EMSA, the fragment corresponding to the *ygeH* promoter region DNA (50 ng) was mixed with a 3 fold excess of competitor DNA (*hlyR* [33]) in a final volume of 20 μl, using the same binding buffer as described above. Increasing concentrations of H-NS–His were added as indicated, and the reaction was incubated at room temperature for 30 min. The samples were resolved, stained and visualized as described above. Purified H-NS–His protein was obtained as described [34], and the *hlyR* DNA was obtained as described by [33].

## Results

### The invasion gene regulator coded by *ygeH* shares significant homology to EilA and HilA proteins

**Figure 1:**
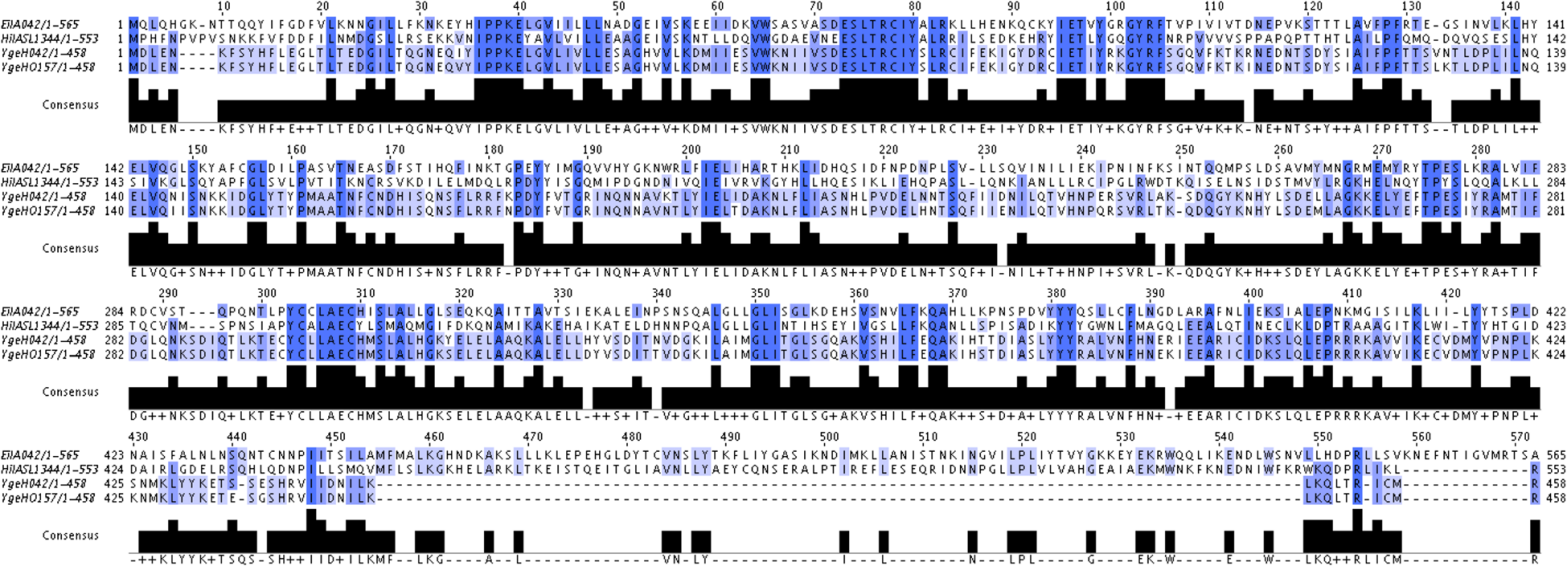
**Alignment showing the amino acid degree of conservation between HilA-like proteins.** Sequence alignment using T-Coffee algorithms (http://www.ebi.ac.uk/Tools/msa/tcoffee/) of the amino acid sequences of the EilA, HilA, YgeH_042_ and YgeH_O157_ proteins [Swiss-Prot: D3H2F7, E1WAC1, D3GSU9, Q8X6H5, respectively]. The different intensities of blue colours indicate the amino acid degree of conservation. The black boxes represent the consensus sequence resulting from the alignment.

The complete genome sequence of *E. coli* 042 strain has led to the identification of two open reading frames encoding two proteins EilA (565 aminoacids) and YgeH (458 aminoacids) that exhibit similarity with the *S.* Typhimurium HilA protein (37% and 29% of similarity with HilA, respectively). In addition to the already reported EilA regulator [19], the putative invasion gene regulator YgeH shows a significant degree of conservation (Figure 1). Whereas EilA appears to be restricted to *E. coli* 042 and other enteroaggregative strains, YgeH is also present in other *E. coli* strains, such as K12, enterohemorrhagic O157:H7, enteroaggregative hemorrhagic O104:H4 and others [18]. In spite of the significant degree of identity at the amino acid level between HilA and YgeH, no information is available about its biological activity, neither regulatory role nor its targets. Thus, we decided to further characterize this latter protein from strain 042.

### EilA and YgeH proteins from *E. coli* strain 042 complement the HilA^−^ phenotype in *S.* Typhimurim strain SV5015

**Figure 2:**
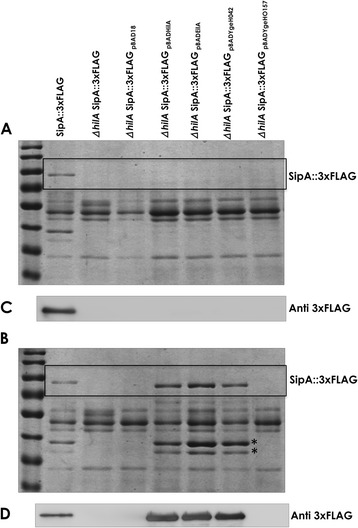
**The HilA-like proteins EilA and YgeH from EAEC 042 are both able to complement** ***in trans*** **the ΔHilA phenotype in** ***S.*** **Typhimurium.** Panel **A** and **B**: SDS-PAGE 10% from whole secreted protein from strains MHS-2 (SV5015 *sipA*::3xFLAG) and MHS-3 (*sipA*::3xFLAG *hilA*) stained with Coomassie brilliant blue. **C** and **D**: Western blots from whole secreted protein from strains MHS-2 and MHS-3. **A** and **C**: Samples obtained from cultures grown in LB medium. **B** and **D**: Samples obtained from cultures grown in LB 0.2% L-arabinose. The SipA::3xFLAG protein was detected using monoclonal anti-flag antibody. The asterisks indicate the expression of other effector proteins according to [36], which are also activated by HilA-like proteins.

We addressed first the question whether the YgeH proteins from strain 042 (YgeH_042_) and from strain O157 (YgeH_O157_) could be functionally equivalent to HilA. To test this, we first constructed a Δ*hilA* mutant of *S.* Typhimurium strain SV5015 (see Methods section). To monitor HilA activity we tested the expression of the *sipA* gene. The *sipA* gene is encoded in the *sic/sip* operon. The SipA effector is secreted by the type III secretion system encoded in the *Salmonella* pathogenicity island 1 (SPI1). Expression and translocation of SipA requires HilA activation of SPI1 operons [12,35]. To detect SipA, a 3xFLAG was fused to its C-terminal end. For the complementation studies, *eilA* and *ygeH* genes from strain 042 and the *ygeH* gene from strain O157 were cloned in the expression vector pBAD18 [26]. Wild-type SV5015SipA (SipA::3xFLAG) and its Δ*hilA* derivative (SV5015 SipA::3xFLAG Δ*hilA*) were transformed with plasmids pBAD18, pBADHilA, pABDEilA, pBADYgeH_042_ and pBADYgeH_O157_. Cultures of the different transformants were grown in LB medium containing carbenicillin. Proteins present in the corresponding supernatants were analyzed by SDS-PAGE and Coomassie Brilliant Blue staining (Figure 2A). Moreover SipA::3xFLAG was immunodetected with anti-flag specific antibodies. As expected, SipA protein could only be detected in wt strain SV5015 SipA::3xFLAG, but not in the corresponding Δ*hilA* derivative (Figures 2C). Alternatively, cultures of these strains were grown in LB medium containing carbenicillin and 0.2% arabinose. Overexpression of EilA, YgeH_042_, and YgeH_O157_ upon arabinose induction was confirmed by qRT-PCR. Upon arabinose inducing conditions, plasmids pBADHilA, pBADEilA, and pBADYgeH_042_ complemented *in trans* the Δ*hilA* mutation (Figure 2B and D). Interestingly, plasmid pBADYgeH_O157_ does not complement Δ*hilA*. The amount of expressed SipA::3xFLAG was very similar in strains harbouring either pBADHilA, pBADEilA or pBADYgeH. Other effector proteins could also be detected in cells expressing any of the three HilA-like modulators (Figure 2B). These results provide evidence for *E. coli* strain 042 EilA or YgeH proteins being able to functionally replace HilA in *Salmonella,* but not for YgeH_O157_. It is noteworthy that, compared to YgeH_042_, YgeH_O157_ presents 22 amino acid substitutions, that might result in loss of protein function (see Discussion section).

### EilA and YgeH_042_ activate *sipA* expression via InvF in *Salmonella*

**Figure 3:**
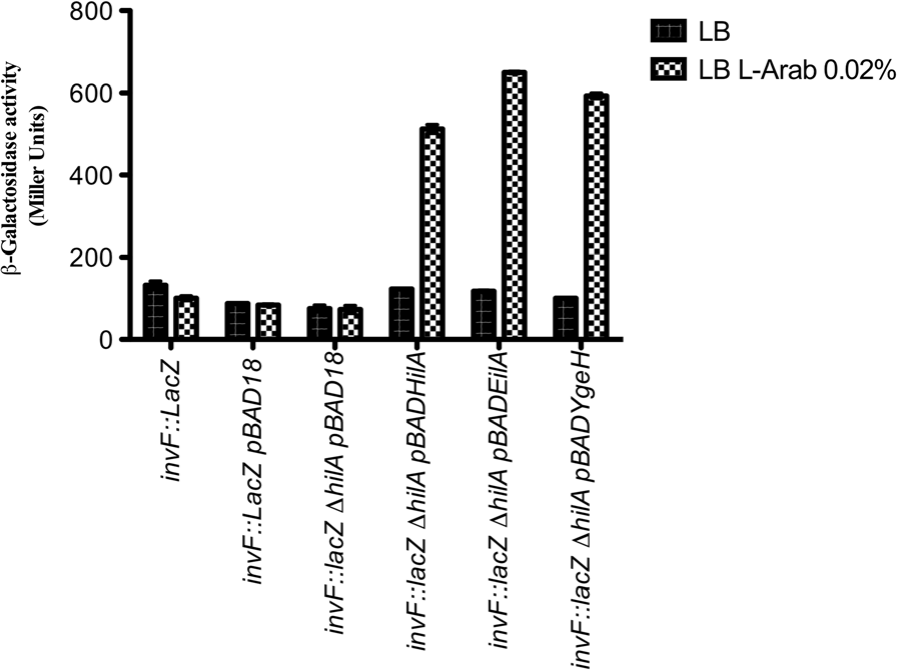
**Expression of EilA and YgeH proteins induces** ***invF*** **expression.** Transcription of *invF* in strains SV5258 and *SV5258hilA* was evaluated by measuring β-galactosidase activity in the different constructs. Samples were obtained from LB CB_100_ or LB CB_100_ L-arabinose 0.02% cultures at the beginning of stationary phase (O.D._600_ of 2.0) at 37°C. Standard errors from three independent experiments are shown.

The regulatory cascade leading to *sipA* expression requires HilA-mediated activation of InvF which, in turn, activates SipA [36]. To check if either EilA or YgeH_042_ would influence *sipA* expression by activating InvF, strain SV5258 (*invF::lacZ* ) was used. A Δ*hilA* derivative was constructed by transducing the Δ*hilA*::Cm allele from strain MH-C1*.* Strain SV5258 was transformed with plasmid pBAD18, and strain SV5258*hilA* was transformed with plasmids pABD18, pBADHilA, pBADEilA and pBADYgeH_042_. The corresponding transformants were grown at 37°C to the beginning of the stationary phase (OD_600_ 2.0) either in LB medium or in LB medium containing 0.02% arabinose. As expected, neither arabinose nor the vector pBAD18 affected *invF* expression in the *hilA*^*+*^ strain (Figure 3). In contrast, in Δ*hilA* mutant strain, the presence of plasmids pBADHilA, pBADEilA or pBADYgeH_042_ drastically increased *invF* transcription when cells were grown in LB medium containing arabinose (Figure 3). Hence, the effect of both EilA and YgeH_042_ influencing *invF* transcription is similar to that of HilA.

### Regulation of *ygeH*_042_ expression

We recently shed light on the role of the nucleoid-associated proteins H-NS and Hha modulating *hilA* expression in *Salmonella* in a temperature-and growth phase-dependent manner. IHF appeared to antagonize H-NS-mediated repression of *hilA* when cells reach the stationary phase of growth [20]. We decided to study the growth conditions influencing *ygeH*_042_ expression and the hypothetical role of the global modulator H-NS on *ygeH*_042_ expression. We tried to construct a transcriptional *ygeH::lacZ* gene fusion in the chromosome of strain 042. Under our working conditions, it was not possible to obtain this fusion in the 042 chromosome. We therefore decided to construct it in strain AAG1 (a Δ*lacZ* derivative from strain MG1655). Expression studies were performed in wt cells and in cells lacking the modulators H-NS and/or IHF at varying temperatures and osmolarities, in exponentially growing cells as well as in cells entering the stationary phase. The most relevant regulatory transcriptional data obtained in strain AAG1 were thereafter validated in strain 042 by detecting YgeH::3xFLAG by qRT-PCR and Western Blotting, as well (see below).

**Figure 4:**
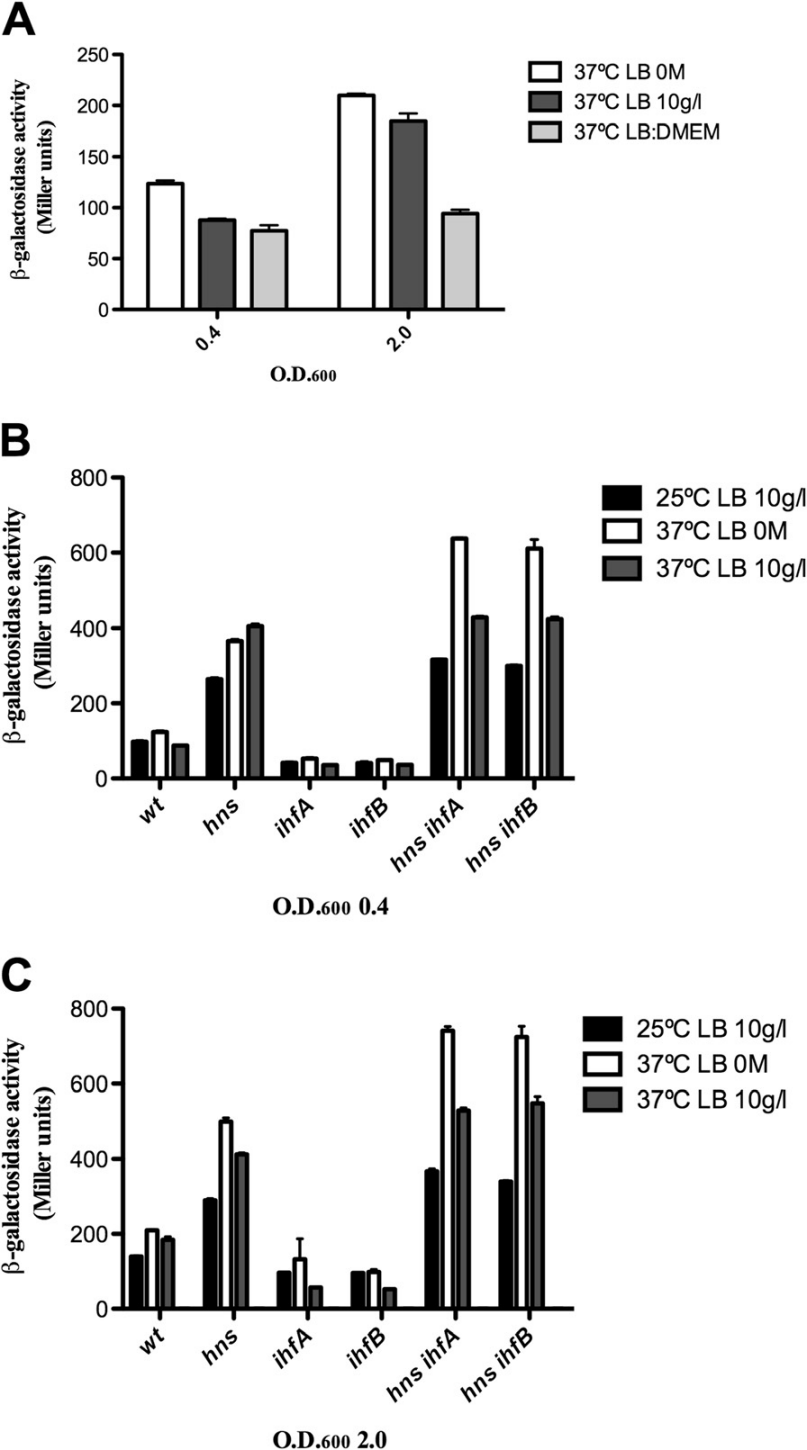
**Effect of growth-phase, temperature,** ***hns*** **and** ***ihf*** **mutations on** ***ygeH::lacZ*** **expression.** β-galactosidase activity of the *ygeh::LacZ* fusion was determined in *E. coli* strain AAG1 **(A)** and in different genetic backgrounds **(B and** **C)** grown under either different temperatures or growth media. Samples were obtained at the log and the onset of the stationary growth phases (O.D._600_ of 0.4 and 2.0, respectively). Standard errors from three independent experiments are shown.

The expression profile of *ygeH*_042_ in different culture condition shows that stationary growth phase influences its expression, moderately increasing it. No effects of growth temperature or osmolarity are apparent (Figure 4A). With respect to the modulators H-NS and IHF, H-NS significantly represses *ygeH*_042_ expression in the different conditions tested, including the onset of the stationary phase. IHF appears to be required for a proper expression of *ygeH*_042_. The fact that deletion of either *ihfA* or *ihfB* only decreases *ygeH*_042_ expression when the H-NS protein is available, suggest that, in the *ygeH*_042_ regulatory region, IHF might antagonize H-NS (Figure 4B and C).

**Figure 5:**
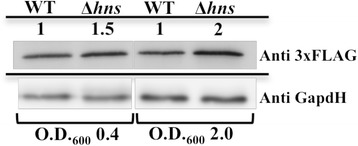
**The H-NS protein represses the** ***ygeH*** **gene at 37°C in strain 042.** Immunodetection of YgeH::3xFLAG in strains 042YgeH3X (YgeH::3xFLAG) and its isogenic mutant derivate 042YgeH3XHNS (YgeH::3XFLAG Δ*hns*). Cultures were grown in LB medium at 37°C and samples were respectively collected at the logarithmic and onset of the stationary phase (OD_600_ of 0.4 and 2.0). Whole cell lysates (5 μg) were resolved by 12.5% SDS-PAGE. The YgeH::3xFLAG protein was detected using the monoclonal anti-FLAG antibody. The GapdH protein was used as a protein loading control in western blot analysis and was detected using the monoclonal anti-GapdH antibody. The values shown under the strain name represent the relative amount of protein found in the mutant strains as compared to the wt strain (set as 1).

To confirm that the observed regulatory effect of H-NS on *ygeH*_042_ expression in strain AGG1 also takes place in strain 042, y*geH*_*042*_ transcription was detected in strains 042 and 042Δ*hns* by both real-time qRT-PCR and YgeH protein immunodetection*.* qRT-PCR shows an upregulation in the Δ*hns* mutant of the +4.5 ± 0.17 compared to wt strain. To detect YgeH_042_, 3xFLAG was fused to its C-terminal end. The YgeH::3xFLAG was immunodectected by using the monoclonal anti-flag antibody. As expected from the results obtained in strain AAG1, expression of *ygeH*_042_ increased in the absence of H-NS (Figure 5).

**Figure 6:**
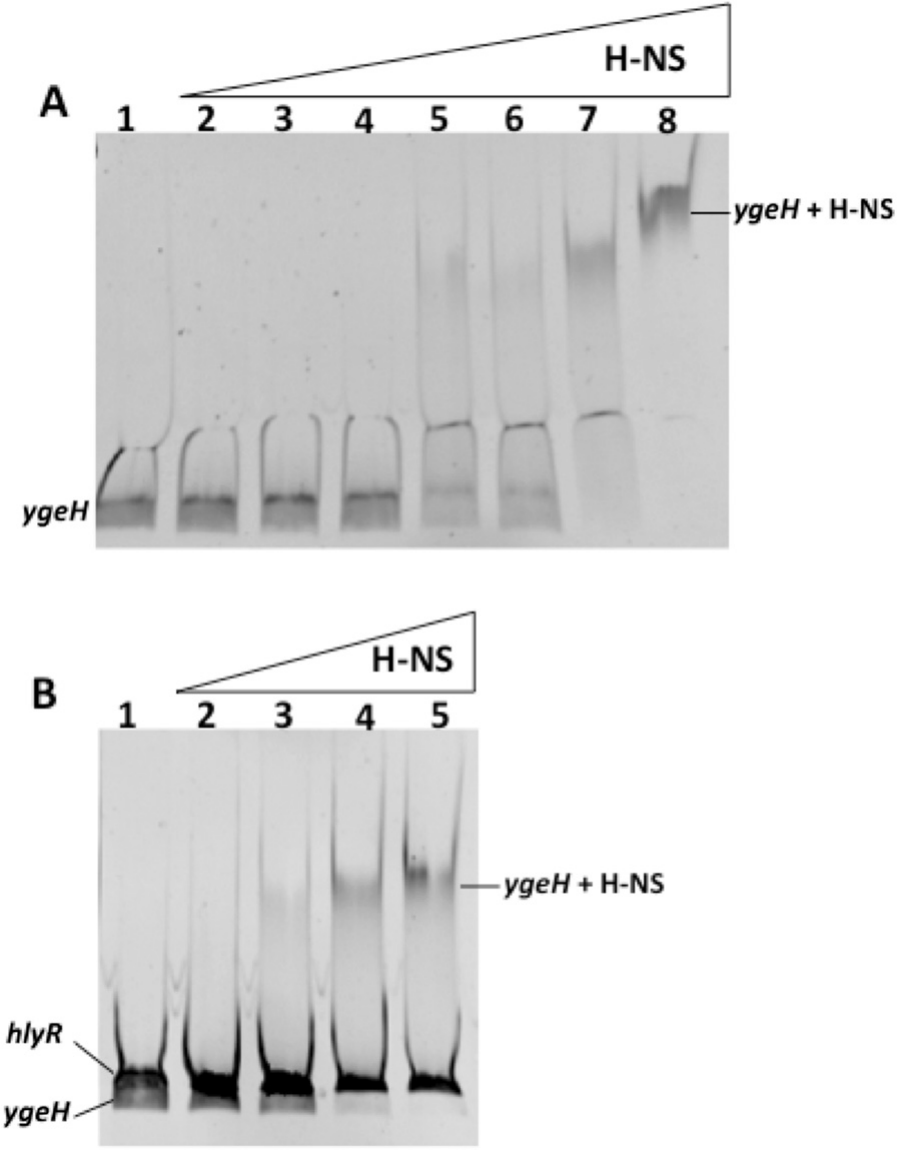
**H-NS-His protein binds to the** ***ygeH*** **regulatory region.** Panel **A**: EMSA using a 228 bp fragment corresponding to the *ygeH* operon promoter region (nucleotides −207 to +21) and H-NS-His protein. 1) No protein, 2–8) increasing concentrations of H-NS-His (0.1, 0.2, 0.5, 1, 1.5, 2 and 4 μM, respectively). Panel **B**: Competitive EMSA using the DNA fragments corresponding to the *ygeH* operon promoter region and an excess of 3 fold of the competitor DNA (*hlyR*), in the presence of increasing concentrations of H-NS. 1) No protein, 2–5) increasing concentrations of H-NS-His (1, 1.5, 2 and 4 μM, respectively).

We studied next if H-NS binds with specificity to *ygeH*_*042*_ regulatory region. A virtual footprinting analysis of this region showed that it includes three putative sequences, predicted to be H-NS binding sites (see Additional file 2). A 228 bp DNA fragment that includes the *ygeH*_*042*_ promoter region was used for EMSA assays. According to the transcriptional data, binding of H-NS could be shown for the selected DNA fragment (Figure 6A). To provide evidence for specificity of binding, a competitive band shift experiment was performed, using both the fragment corresponding to the *ygeH*_*042*_ regulatory region, and a DNA fragment, corresponding to the regulatory region of the *hly* operon (407 bp). H-NS does not show specific binding to this fragment [33]. The competitive band shift assay showed that H-NS preferentially binds to the *ygeH*_*042*_ regulatory region (Figure 6B).

### YgeH upregulates ETT2 determinants

**Figure 7:**
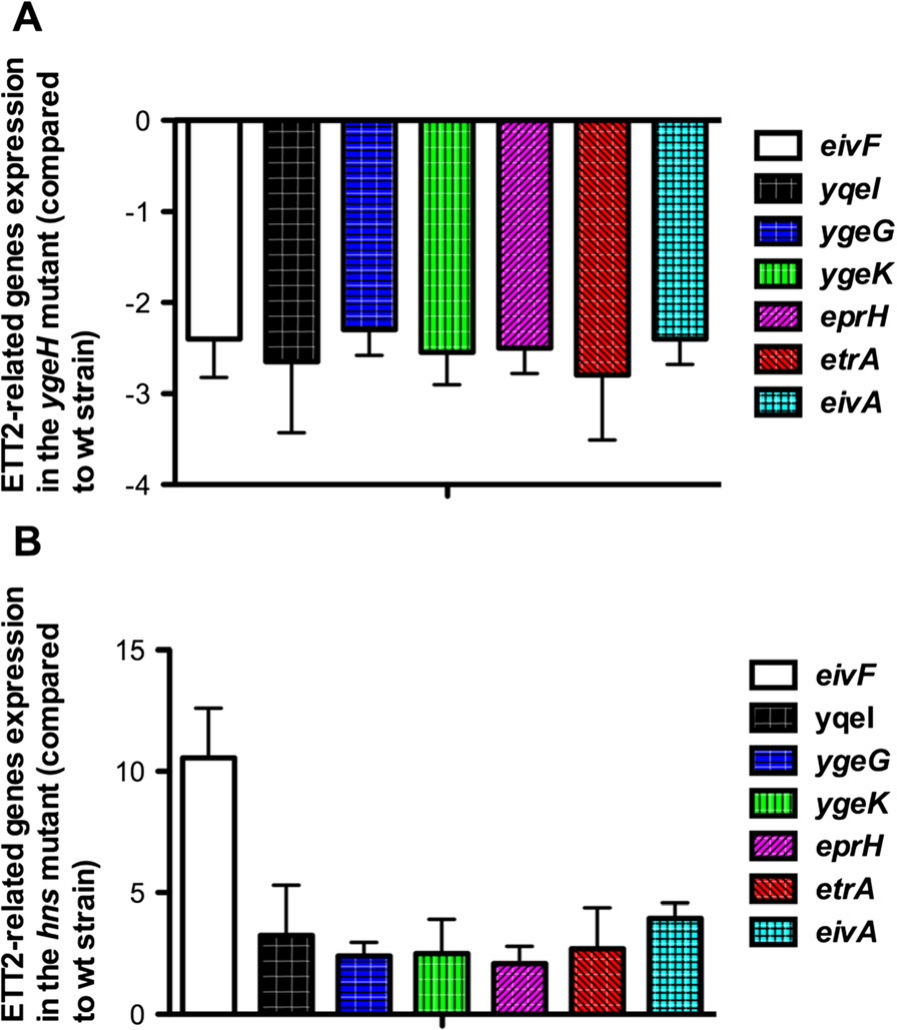
**Effect of ∆** ***ygeH*** **and ∆** ***hns*** **mutations on the expression of ETT2 genes from EAEC 042.** Expression of ETT2-related genes was determined by reverse transcription and qRT-PCR. Data show the transcription levels of ETT2-related genes in bacteria cultured in LB broth to early-stationary phase (O.D._600_ of 2.0). As internal standard the *gapA* gene was used. Data are means of three independent experiments. Standard deviations are shown. Panel **A**: Relative expression of the ∆*ygeH* mutant relative to wt strain. Panel **B**: Relative expression of the ∆*hns* mutant compared to wt strain.

Taking into account that YgeH is encoded in the ETT2 island, we decided to test the effect of YgeH depletion on expression of different ETT2 encoded genes. Genes selected corresponded to most of the ETT2-encoded operons, and were the *eivF* gene, encoding an InvF-like protein, *eivA, etrA, eprH, ygeG, ygeK* and *yqeI* (locus tag: EC042_3070, EC042_3060, EC042_3059, EC042_3049, EC042_3053, EC042_3045, respectively). Expression of these genes was first monitored by qRT-PCR in the wt 042 strain and in the *ygeH*_*042*_ single mutant. As expected from YgeH being a transcriptional activator, depletion of *ygeH* resulted in downregulation of the different ETT2 genes (Figure 7A). Taking into account that *ygeH* expression is increased in an *hns* mutant, we decided to further investigate whether, as a response to reduced H-NS levels, ETT2 genes are also upregulated in a ∆*hns* mutant. As expected, all the ETT2 genes tested are upregulated in strain 042 Δ*hns* (Figure 7B). Hence, expression of ETT2 genetic determinants is subjected to H-NS-mediated repression via YgeH.

## Discussion

In this report we provide evidence for the close relationship between the *E. coli* 042 YgeH protein, the EilA regulator and the *Salmonella* regulator HilA. Genomic studies have shown that the ETT2 island is widespread in *Escherichia* and *Shigella* strains [18,37,38], although in many of them the gene cluster is either disrupted or incomplete [18]. The YgeH protein, which shares significant similarity to the SPI1 modulator HilA, is present in several of the strains analyzed as, for instance, 042, O157:H7 or MG1655. We show here that the YgeH protein is functional in strain 042. Furthermore, the YgeH protein, as well as the already characterized EilA protein, is able to complement the ∆*hilA* mutation in *Salmonella*. All of them target the SPI1 gene *invF* which, in turn, activates the expression of effectors such as SipA. These results support the close functional and structural relationship between these proteins and suggest a common origin. Remarkably, whereas either YgeH_042_ or EilA complement the *hilA* mutation, YgeH_O157_ does not. The amino acid sequence of YgeH_042_ and of YgeH_O157_ differs in several amino acid residues, and these differences may be underlying loss of protein function.

Unlike *eilA* gene [19], *ygeH* is expressed when 042 cells grow in LB medium. Unlike *hilA*, significant expression levels are detected both at the exponential and stationary growth phases. Only a moderate increase in transcription is apparent at the onset of the stationary phase. When growing in LB medium, neither temperature nor osmolarity are critical factors altering *ygeH*_042_ expression. Remarkably, H-NS appears as a repressor of *ygeH*_*042*_ expression. Under all growth conditions tested, the *hns* mutant from strain 042 showed an upregulated expression of *ygeH*. Whereas these data show that H-NS is a repressor of *ygeH*_*042*_, it is also apparent that environmental conditions leading to a full induction of *ygeH*_*042*_ (i.e., environmental conditions which would result in similar expression levels of *ygeH*_*042*_ in both wt 042 strain and the *hns* mutant) are not among those used in this work. H-NS targeting *ygeH*_*042*_ is also supported by the EMSA assays in the presence of a DNA fragment including the *ygeH* regulatory region and a competitor DNA molecule. Altogether, these data show that, when coping with the appropriate environmental conditions, 042 cells must be able to significantly increase YgeH levels by counteracting H-NS silencing.

A question to be addressed is the biological role of the either complete or incomplete ETT2 determinants in different *E. coli* isolates, ranging from MG1655 to O157:H7. Some previous data together with the results presented in this paper may help to clarify this. In a previous report [39], *ygeH, etrA and eivF* genes from the ETT2 genetic element from *E. coli* O157:H7 were mutagenized, and the effect of the corresponding mutant alleles on expression of the LEE (locus of enterocyte effacement) pathogenicity island was studied. Whereas disruption of the *ygeH*_O157_ gene did not influence LEE expression (and hence it was not further characterized), the *etrA* and *eivF* mutant alleles were found to significantly enhance secretion of proteins encoded by the LEE. Transcription of several LEE genes was altered in these mutants. We show in this report that, unlike YgeH_042_, YgeH_O157_ fails to complement the *ΔhilA* mutation. The production of a non-functional YgeH protein in *E. coli* strain O157 may be underlying the fact that, whereas *etrA* and *eivF* mutants of strain O157 influence LEE expression, *ygeH* mutants do not [39]. Hence, mutational attrition that occurs in some genomic islands may also affect some of the encoded modulators, thus resulting in a complex array of functional and non-functional genes that may significantly increase the genomic plasticity of those strains that incorporate the corresponding genomic islands.

## Conclusions

YgeH and EilA proteins can functionally replace HilA in *Salmonella. E. coli* 042 YgeH protein expression is similar when cells grow in a wide variety of lab culture conditions. Whereas YgeH expression upregulates expression of ETT2 genes, H-NS downregulates their expression. As exemplified by YgeH_O157_ protein, some of the YgeH modulators encoded in ETT2 islands may be non-functional. Hence, mutational attrition identified in the ETT2 cluster is not restricted to YgeH-modulated genes, but may also affect the modulator itself.

## Additional files

Additional file 1
**Sequence of the oligonucleotides utilized in this study.**
Additional file 2
**Putative H-NS binding sites found in the regulatory region of the** ***ygeH***_**042**_ **gene.**

## Abbreviations

SPI1: *Salmonella* pathogenicity island 1
TTSS: Type 3 secretion system
ETT2: *Escherichia coli* type 3 secretion system 2
LEE: Locus of enterocyte effacement
Km: Kanamycin
CB: Carbenicillin
Cm: Choramphenicol
Sm: Streptomycin
Tc: Tetracycline
TCA: Trichloroacetic acid
