# Supplementary material for: HilA-like regulators in Escherichia coli pathotypes: the YgeH protein from the enteroaggregative strain 042
Source: BMC Microbiol. 2014 Oct 25;14:268. doi: 10.1186/s12866-014-0268-5 (PMC4210603; doi:10.1186/s12866-014-0268-5)
Supplement: Additional file 1 — Sequence of the oligonucleotides utilized in this study. [file 12866_2014_268_MOESM1_ESM.pdf]

Additional file 1: Sequence of the oligonucleotides utilized in this study.

**Name**	**Sequence (5´ - 3´)**
**CATC1**	CCTTGTCGCCTTGCGTATAA
**CATC2**	CCTACCTGTGACGGAAGATC
**IHFAP1**	ATGGCGCTTACAAAAGCTGAAATGTCAGAATATCTGTTTGATGTGTAGGCTGGAGCTGCTTC
**IHFAP2**	TTACTCTTCTTTGGGCGAAGCGTTTTCGACCCGGCTCTTTAACATATGAATATCCTCCTTAGT
**IHFAP1UP**	GTGTAGAGGCATTAAAAGAGCG
**IHFAP2DOWN**	CAAAAGGTCAGCATGTTATCC
**IHFBP1**	ATGACCAAGTCAGAATTGATTGAAAGACTTGCAACCCAGCAAGTGTAGGCTGGAGCTGCTTC
**IHFBP2**	TTAACCGTAAATATTGGCGCGATCGCGCAGTTCTTTACCCGGCATATGAATATCCTCCTTAGT
**IHFBP1UP**	GCAGCCAATTTGCCTTTAAGG
**IHFBP2DOWN**	CCGTCGTTATCTTCATAGAC
**SIPA3XP1**	TCGGGTTATTACTACCGTTGATGGCTTGCACATGCAGCGTGACTACAAAGACCATGACGG
**SIPA3XP2**	AATATCCATATTCATCGCATCTTTCCCGGTTAATTAACGCCATATGAATATCCTCCTTAG
**SIPA3XP1UP**	CGTTGAAAGCGCAATGTAG
**SIPA3XP2DOWN**	CATATATTGCAGCCATACTC
**HNS042P1**	ATGAGCGAAGCACTTAAAATTCTGAACAACATCCGTACTCTTGTGTAGGCTGGAGCTGCTTC
**HNS042P2**	TTATTGCTTGATCAGGAAATCGTCGAGGGATTTACCTTGCCCATATGAATATCCTCCTTAGT
**HNS042P1UP**	CCACCCCAATATAAGTTTGAG
**HNS042P2DOWN**	GGGATTTTAAGCAAGTGCAATC
**YGEH042P1**	ATGGACTTAGAAAATAAATTCTCATATCATTTTCTTGAGGGATTAACGCTCGTGTAGGCTGGAGCTGCTTC
**YGEH042P2**	TTATCGCATACAAATTCTTGTCAGTTGCTTTAGTTTCAAAATGTTGTCAATCATATGAATATCCTCCTTAGT
**YGEH042P1UP**	CTCATATCATTTTCTTGAGGG
**YGEH042P2DOWN**	CTTGTCAGTTGCTTTAGTTTC
**YGEHBAMHI5´**	CGGGATCCGCATTACAATCATTTGGTTAAAATTGC
**YGEHBAMHI33**	CGCGGATCCATTTTCTAAGTCCAT
**EILAKPNI5´**	GGGGTACCGAATAGAGAAAACGGAAGGAGCGTTCTATG
**YGEHKPNI5´**	GGGGTACCCAATTTTTTCATAGAGGTTAACTAATG
**EILAHINDIII3´**	CCCAAGCTTTTACGCAGAAGTACGCATCACACC

**YGEHHINDIII3´**	CCCAAGCTTTTATCGCATACAAATTCTTGTCAG
**PBADFW**	GCTATGCCATAGCATTTTTATCC
**PBADRV**	GATTTAATCTGTATCAGGCTG
**HILABADFW**	CGGAATTCGTAAGAGAATACACTATTATCATGCCA
**HILABADRV**	GCTCTAGATTACCGTAATTTAATCAAGCGGGG
**KT**	CGGCCACAGTCGATGAATCC
**K2**	CGGTGCCCTGAATGAACTGC
**YGEHRTFW**	TGGGACTGATAACTGGTCTGT
**YGEHRTRV**	AGCGGGTTAGGCACATACAT
**EIVFRTFW**	CCAGGCTTCACTTGCAACTT
**EIVFRTRV**	GCTCATTGACCGAAGCCAAA
**GAPA042FW**	TTTCCGTGCTGCTCAGAAAC
**GAPA042RV**	TTTCCGTGCTGCTCAGAAAC
**YGEIRTFW**	TTTCGCGCTGTTGGTGATAC
**YGEIRTRV**	CCCCGCATTACCTTTTACGG
**HLYSAL/ECO-5´**	CAGACCACACCTGGAAAAAC
**HLYSAL/HIND3´**	CGGCTTCACTGCGAAATTCA
**YGEGRTFW**	GCTGCATGGCTGTCGTCAG
**YGEGRTRV**	CGCAGGTTCCCCTACGGTT
**YGEHBAMHI5´**	CGGGATCCGCATTACAATCATTTGGTTAAAATTGC
**YGEHBAMHI33**	CGCGGATCCATTTTCTAAGTCCAT
**YGEKRTFW**	GTTTCAGATCAGCAGCCGTT
**YGEKRTRV**	TGAGTTCGGACCCCATCATT
**EPRHRTFW**	TTGGATGAAGCTGTCTGGGT
**EPRHRTRV**	CCGTTGCTTACTCACTGTCAG
**ETRARTFW**	CGATTTGGTTGCGTTGGTTG
**ETRARTRV**	TCGAAACCAGGAAATGCGTT
**EIVARTFW**	CGGTGGGCTATCAGTAGGAG
**EIVARTRV**	TACTTAGCGCGAGGACACAT
**YGEHO157XBAFW**	GCTCTAGACAATTTTTTCATAGAGGTTAACTAATG

**YGEHO157HINDRV**	CCCAAGCTTTTATCGCATACAAATTCTCGT
