# Supplementary material for: HilA-like regulators in Escherichia coli pathotypes: the YgeH protein from the enteroaggregative strain 042
Source: BMC Microbiol. 2014 Oct 25;14:268. doi: 10.1186/s12866-014-0268-5 (PMC4210603; doi:10.1186/s12866-014-0268-5)
Supplement: Additional file 2 — Putative H-NS binding sites found in the regulatory region of the ygeH 042 gene. [file 12866_2014_268_MOESM2_ESM.pdf]

Additional file 2: Putative H-NS binding sites found in the regulatory region of the *ygeH*$_{042}$ gene.

5´GCATTACAATCATTTGGTTAAAATTGCTGGTTATAGTGCAGTTAACCACTATGACCATCTTCTCTTA

TGTTTTCTTAAATCAACATCTTAAAACAGTTAAGAACACACTATAAATTATTATTTTTCAAC**GCGTTAGA**

GAACAATCT**ATTTTATCAA**AATTGAATAAGGCTATTTCAGATTACATCACTGAATTCCT**AAGATAAATT**A

G**TGAACA**CAACCATAGATATGTCGA**ATGTAAACT**CCTATA**G**CATTAATGACACATAAATATGAATAGCCA
  -35 **-10** **+1**

CAATTTCTATTGCTTATGCAGGATGCAAGAAACCAATTTTTTCATAGAGGTTAACTA**ATG** 3´

The putative H-NS-binding sites were determined using Virtual Footprint software (http://prodoric.tu-bs.de/vfp/). The *ygeH* promoter (GenBank accession, gene locus tag EC042_3050) spanning from positions -247 to +90 (relative to the transcriptional start site) was subjected to analysis using the position weight matrix H-NS from *E. coli* K-12 and the best three matches are shown in red. The -10 and -35 boxes are shown in bold. The transcriptional start site and the translational start site are represented in blue and green, respectively.
